# Structural insights into the calcium dependence of Stig cyclases†

**DOI:** 10.1039/c9ra00960d

**Published:** 2019-04-30

**Authors:** Xueke Tang, Jing Xue, Yunyun Yang, Tzu-Ping Ko, Chin-Yu Chen, Longhai Dai, Rey-Ting Guo, Yonghui Zhang, Chun-Chi Chen

**Affiliations:** School of Life Sciences, University of Science and Technology of China Hefei 230026 China; State Key Laboratory of Biocatalysis and Enzyme Engineering, Hubei Collaborative Innovation Center for Green Transformation of Bio-Resources, Hubei Key Laboratory of Industrial Biotechnology, School of Life Sciences, Hubei University Wuhan 430062 China ccckate0722@gmail.com; College of Biotechnology, Tianjin University of Science and Technology Tianjin 300457 China; School of Pharmaceutical Sciences, Tsinghua University Beijing 100084 China zhangyonghui@tsinghua.edu.cn; Institute of Biological Chemistry, Academia Sinica Taipei 11529 Taiwan; Industrial Enzymes National Engineering Laboratory, Tianjin Institute of Industrial Biotechnology, Chinese Academy of Sciences Tianjin 300308 China

## Abstract

The Stig cyclases from *Stigonematalean cyanobacteria* are classified as a novel type of calcium-dependent cyclases which catalyze an uncommon reaction cascade comprising Cope rearrangement, 6-*exo*-trig cyclization, and electrophilic aromatic substitution. Previously we found two calcium ions near the substrate-binding pocket. The calcium-coordinating residues are conserved in all Stig cyclases. In the present study, we use site-directed mutagenesis to investigate the role of calcium coordination. By individually mutating the coordinating residues in either of the Ca^2+^-binding sites to alanine, the enzyme activity is significantly reduced, suggesting that the presence of Ca^2+^ in both sites is essential for catalysis. Furthermore, the crystal structure of N137A, in which the Ca^2+^-binding N137 is replaced by Ala, shows significant local conformational changes, resulting in a squeezed substrate-binding pocket that makes substrate entry ineffective. In conclusion, calcium coordination is important in setting up the structural elements for catalysis. These results add to the fundamental understanding of the mechanism of action of the calcium-dependent Stig cyclases.

## Abstract 2

Crystal structure of a calcium-depleted Stig cyclase revealing the mechanism of metal-dependence of enzyme activity.

## Graphical abstract

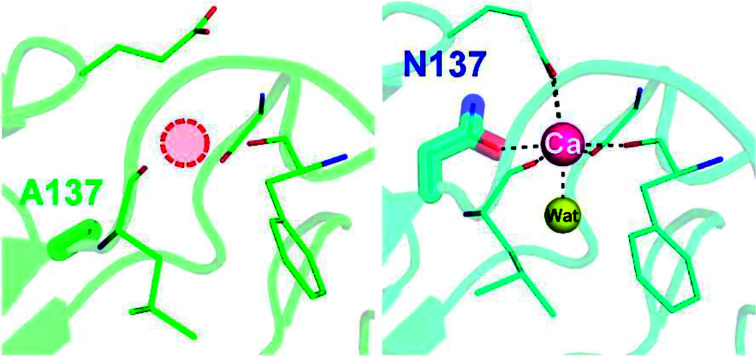

Stig cyclases from *Stigonematalean cyanobacteria* are a new family of enzymes, which catalyze an unusual cascade of carbon–carbon bond forming reactions comprising three steps: Cope rearrangement, 6-*exo*-trig cyclization, and electrophilic aromatic substitution,^1–3^ enabling the transformation of a geranylated indolenine to generate a variety of hapalindole-type indole alkaloids. The enzyme products have attracted much attention due to their highly functionalized stereo- and regio-chemically diverse polycyclic ring system (ESI, Scheme 1†), as well as their broad spectrum of biological profiles.^4^ In addition, enzyme catalyzed Cope rearrangement is very rarely found in nature.^5,6^ Thus it is of great importance to unravel the underlying mechanism of Stig cyclases.

Several Stig cyclases have been identified and their functions characterized.^1–3,7^ While these enzymes share a high protein sequence identity (>60%), their catalytic products exhibit significant variations in C-ring closure configuration and stereochemistry at multiple chiral centers. Based on the catalytic products, Stig cyclases are classified into three subfamilies (ESI, Scheme 1†).^2^ Recently, we and the other group reported the crystal structures of five Stig cyclases belonging to subfamily 1 and 2.^8,9^ These enzymes fold into two β-sheets that pack against each other to form a β-sandwich. The closest structures are the β-sandwich type of non-catalytic carbohydrate-binding motifs (CBMs). Furthermore, we determined several complex structures of Stig cyclases with a series of substrate mimics.^8^ These ligands were found to bind to a hydrophobic terminal cavity, which is surrounded by several extensive loops at the distal end of the larger β-sheet. The strictly conserved catalytic residue D214 (D215 in subfamily 2) plays a key role in binding substrate and triggering catalytic cascades. The adjacent Y89 (Y90 in subfamily 2), also strictly conserved in all Stig cyclases so far identified, forms a hydrogen bond to D214 *via* side chain hydroxyl group. Substitution of Y89 with a Phe resulted in more than 90% loss of activity, suggesting a significant role of the D214-Y89 pair in the catalysis.

Stig cyclases represent a novel group of cyclase whose activity depends on the binding of Ca^2+^ ion. A previous report showed that EDTA treatment abrogates the Stig cyclase activity.^3^ Subsequently, in crystal structures, two Ca^2+^ ions (Ca1 and Ca2) were found near the substrate-binding pocket.^8,9^ The Ca-coordinating residues are conserved in all Stig cyclases, suggesting the essential role of the metal ions (Fig. 1A). Structural comparison shows that the Ca2 binding site is conserved in both Stig cyclases and β-sandwich type CBMs, but the location of Ca1 is a unique feature of Stig cyclases (Fig. 1B). While the presence of calcium ions has been related to the protein stability of the CBMs,^10,11^ the underlying mechanism of calcium dependence of Stig cyclase remains to be explored.

**Fig. 1:**
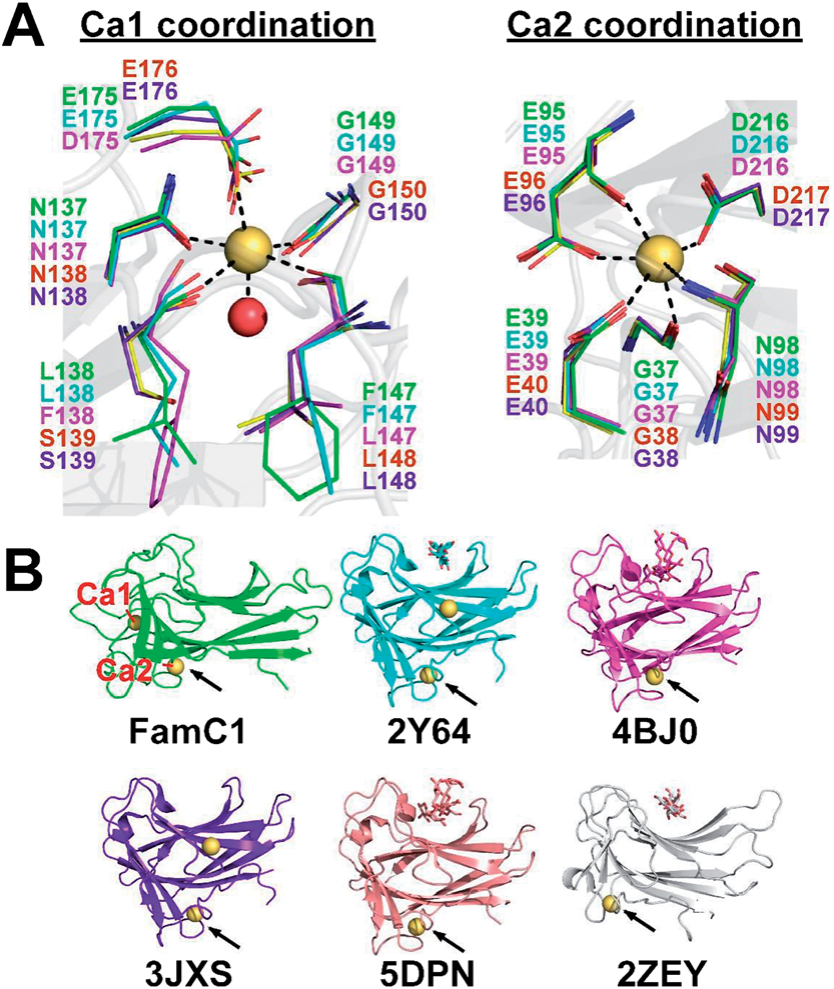
Calcium-binding sites of Stig cyclases and structurally similar CBMs. (A) The coordinating residues to Ca1 and Ca2 in Stig cyclases (subfamily 1: FamC1, green; FilC1, cyan; HpiU5, magenta; and subfamily 2: FimC5, yellow; FisC, purple) are shown as thin sticks. The protein fold of FamC1 (gray cartoon), the metal ions (orange spheres), and a water molecule (red sphere) in the structure are also shown. (B) The overall protein structures are shown as cartoon models, and the bound calcium ions as yellow spheres. Polysaccharides bound to the CBM structures are shown as stick models. The corresponding PDB ID is shown below each structure. Ca1 and Ca2 of the FamC1 structure are labeled, and the Ca2-equivalent site in each CBM structure is indicated by an arrow.

There are two Ca^2+^-binding sites in FamC1 (Fig. 1). To investigate the role of individual calcium ion in a Stig cyclase, the six FamC1 residues whose side chains contribute to Ca^2+^-coordination were mutated to Ala and each variant's activity was examined by measuring the amounts of the final product. As shown in Fig. 2, the variant N137A, E175A, and N137A/E175A exhibited 2.13%, 33.4%, and 2.3% residual activity, respectively. These results suggest that Ca1 is required for FamC1 to catalyze product formation. Similarly, mutating the Ca2-coordinating E98 and D216 to Ala resulted in deleterious effects that less than 3% activity was detected. E39A and N95A also exhibited significantly lower product formation rate, that 12.6% and 60% residual activity was detected, respectively. The variant containing four Ala substitutions exhibited only 1.4% activity. Compared to the other variants, E175A and N95A exhibited milder effects on the FamC1 activity, presumably due to their less contribution to calcium coordination. Alternative factors such as water molecules may replace the protein residues in Ca^2+^-coordination. Overall, these results indicate that both Ca1 and Ca2 coordination systems are critical for FamC1 activity.

**Fig. 2:**
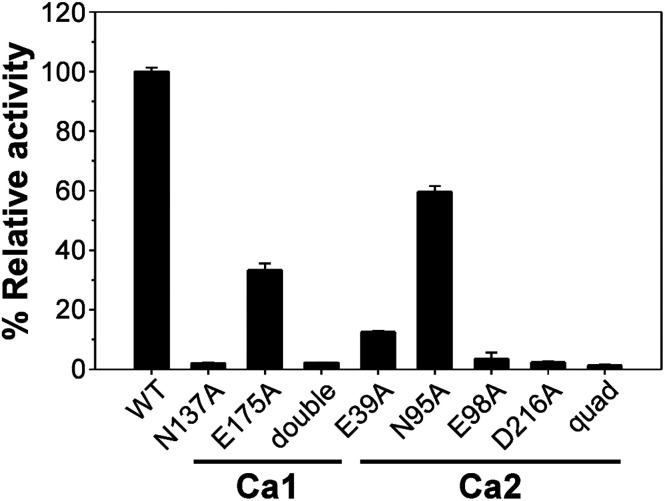
Enzyme activity comparison of FamC1 and the variants. Recombinant proteins of wild-type FamC1 and the variant were subjected to one-pot reactivity analysis to estimate the rate of cyclized product production. The amounts of product of the variants are each measured and presented as percentages of the wild type enzyme. Each protein was examined in triplicate and the average ± SD was calculated.

To investigate the effects of calcium depletion on protein structure, the recombinant protein of all Ala variants were subjected to crystal screening. However, only FamC1 N137A was crystallized successfully, which diffracted X rays to resolution as high as 1.48 Å (Table S1†). The absence of Ca^2+^ may influence overall structure stability and/or flexibility, posing difficulty to the other variant to be crystallized. The N137A crystal belongs to the trigonal space group *P*3_1_21, isomorphous to the wild type FamC1 crystal in our previous study,^8^ and the structure was solved by straightforward molecular replacement. Judging from the electron density maps, residue 137 had been successfully mutated to Ala (Fig. S1A†). Besides, Ca1 was not observed in the N137A crystal, but Ca2 binding site remained intact (Fig. 3A and S1†). These results suggest that the mutation specifically disabled the Ca1 binding. The N137A variant structure deviates from the wild type by a Cα-RMSD value of 0.17 Å (Fig. 3A and S2†), suggesting the mutation had minimal effects on overall protein fold and homodimeric organization.

**Fig. 3:**
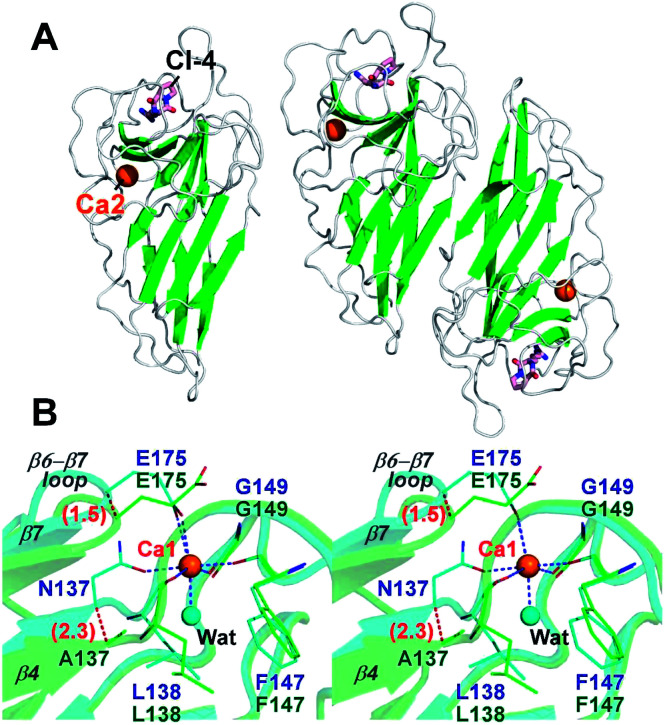
Overall structure and Ca1-binding site of the N137A variant. (A) Crystal structure of the N137A variant is shown as a cartoon model. Both monomeric (left panel) and dimeric (right panel) organization of the protein are displayed. (B) Stereo-view of superimposed wild-type FamC1 (cyan) and N137A (green). Ca1-coordinating residues of the wild-type and the equivalent residues of N137A are shown as thin stick models; the bound ligand of CI-4 and calcium ions as thick stick and sphere models; and a water molecule which coordinates Ca1 in wild-type FamC1 as a cyan sphere. Blue dashed lines indicate the metal-ion coordinate bonds. Numbers in parentheses indicate distance (unit Å).

Notwithstanding, apparent conformational changes were observed around the Ca1-binding region (Fig. 3B). While residue 147 and 149 remained at the same positions, residue 137, 138 and 175 were slightly shifted away from that in the wild-type enzyme (Fig. 3B). The Cα positions of the residue 137 and 175 in N137A variant deviate from those in the wild-type structure by 2.3 Å and 1.5 Å, respectively. E175, which coordinates to the Ca1 ion *via* its carboxyl group in wild-type FamC1, turns outwards from the central position while not coordinating Ca1. These conformational changes lead to deviations of the β4 and β7 strands and the β6–β7 loop. Clearly, these structural elements are held in position by Ca1-coordination in FamC1, and their locations may wobble considerably in the absence of this metal ion in N137A.

In our previous study, a cyclo-dipeptide CI-4 was observed in the wild-type FamC1 crystal. This unexpected ligand was most likely derived from the crystallization solution, and its identity has been confirmed by crystal soaking experiments.^8^ The guanidino moiety of CI-4 was hydrogen bonded to the catalytic residue D214, resembling the indole N of the substrate mimics. The N137A crystal was grown in the same crystallization condition and one molecule of CI-4 was found in the equivalent position (Fig. 3A and S2†). Notably, the Arg side chain of CI-4 in N137A variant turns to the other direction so that the guanidino group is no longer within the hydrogen bonding distance to the D214 side chain (Fig. 4A). However, residue Y89 is positioned between CI-4 and D214 (A-pose), and its hydroxyl group is within H-bond distance to both CI-4's guanidino group and D214's carboxyl group (Fig. 4A, upper panel).

**Fig. 4:**
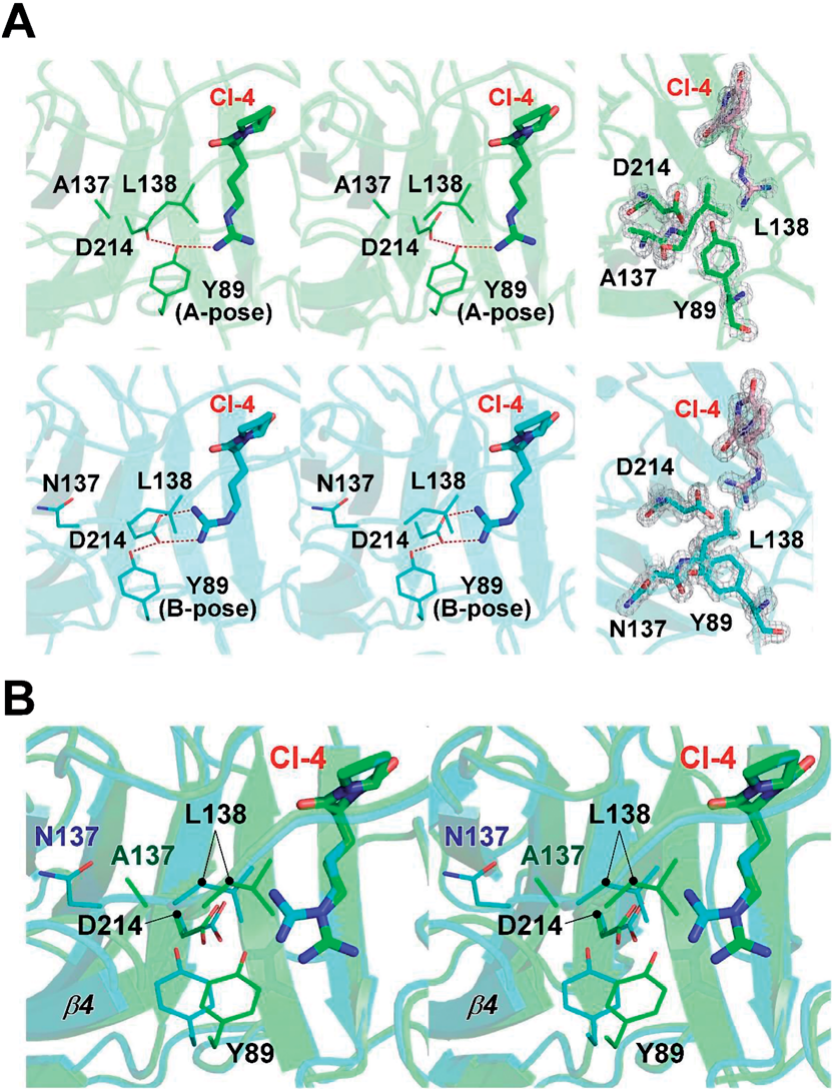
Active site interaction network in the FamC1 structures. (A) Stereo-view of partial CI-4-interacting network in FamC1 structures. The 137 residue, L138, the D214-Y89 pair, and CI-4 in the N137 variant (green) and wild-type FamC1 (cyan) are displayed. The 2*F*_o_ − *F*_c_ electron density maps of these residues and ligands are contoured at 2.0*σ* and shown in the right panel. Y89 in two poses are indicated. (B) Stereo-view of superimposed structures of N137 and wild-type FamC1 is shown with the same color scheme and features as in (A).

In the wild-type FamC1 structure, Y89 took a different pose (B-pose) to yield a space for D214 to bind to the CI-4's guanidino moiety (Fig. 4A, lower panel). No other condition was found effective to obtain FamC1 crystal, and thus the FamC1 structure always harbors CI-4 and Y89 in B-pose. We therefore turned to the other subfamily 1 members (FilC1 and HpiU5) and found that Y89 is in either A- or B- pose in the substrate-free conditions but always has the B-pose in the presence of substrate mimics (Fig. S3†). Based on these analyses, Y89 is mobile in apo-form enzymes, but pushed away upon substrate binding. In N137A, the L138-containing β4–β5 loop and the β4 strand are freed from the Ca1-mediated pulling effects and move towards the substrate-binding pocket (Fig. 4B). As a result, L138-mediated hydrophobic force may hinder the interactions between D214 and the hydrophilic moiety of the substrate, eventually leading to inefficient catalysis. These results provide a reasonable explanation for the lower activity in the Ca1-depleted variant.

## Conclusions

In summary, Stig cyclases make a novel family of calcium-dependent cyclases that catalyze an unusual reaction cascade. The enzyme contains two Ca^2+^-binding sites. In this study, we designed and carried out site-directed mutagenesis studies to clearly demonstrate that both calcium ions are required for the enzyme's activity. More importantly, we solved the crystal structure of the N137A variant of FamC1, in which Ca1-binding was specifically disabled, to reveal the molecular mechanism of calcium-dependence. Structural analyses show that substrate binding to the key catalytic residue D214 might be disrupted due to the lack of Ca1-coordination. Although calcium-demanding proteins are well-documented in intracellular signaling and carbohydrate binding,^12,13^ limited information is available about enzymes whose activity depends on calcium binding.^14,15^ These results provide important guidance to fundamentally understand the calcium dependence of Stig cyclases.

## Author contributions

XT and JX constructed, purified, and crystallized recombinant proteins; YY and LD examined enzyme activity; TPK, CYC, and RTG collected diffraction data, determined and analyzed crystal structure; RTG, YZ and CCC designed the study and prepared the manuscript.

## Conflicts of interest

The authors claim no conflict of interest.

## Supplementary Material

RA-009-C9RA00960D-s001
