# Supplementary material for: Structural insights into the calcium dependence of Stig cyclases
Source: RSC Adv. 2019 Apr 30;9(23):13182–5. doi: 10.1039/c9ra00960d (PMC9063808; doi:10.1039/c9ra00960d)
Supplement: RA-009-C9RA00960D-s001 [file RA-009-C9RA00960D-s001.pdf]

Electronic Supplementary Material (ESI) for RSC Advances.
This journal is © The Royal Society of Chemistry 2019

Electronic supplementary information

Structural insights to the calcium dependence of Stig cyclases

Xueke Tang[a]†, Jing Xue[b,c]†, Yunyun Yang[d], Tzu-Ping Ko[e], Chin-Yu Chen[b], Longhai Dai[b], Rey-Ting Guo[b,f], Yonghui Zhang[d]**, Chun-Chi Chen[b]**

[a]School of Life Science, University of Science and Technology of China, Anhui 230026, China.

[b]State Key Laboratory of Biocatalysis and Enzyme Engineering, Hubei Collaborative Innovation Center for Green Transformation of Bio-Resources, Hubei Key Laboratory of Industrial Biotechnology, School of Life Sciences, Hubei University, Wuhan, 430062, China

[c]College of Biotechnology, Tianjin University of Science and Technology, Tianjin 300457, China

[d]School of Pharmaceutical Sciences, Tsinghua University, Beijing 100084, China.

[e]Institute of Biological Chemistry, Academia Sinica, Taipei 11529, Taiwan

[f]Industrial Enzymes National Engineering Laboratory, Tianjin Institute of Industrial Biotechnology, Chinese Academy of Sciences, Tianjin 300308, China.

*Corresponding e-mail: ccckate0722@gmail.com (CCC);

zhangyonghui@tsinghua.edu.cn (ZY)

†These authors contributed equally to this work.

Experimental

Site-directed mutagenesis

FamC1 variants were prepared by using QuickChange site-directed mutagenesis kit (Agilent Technologies, Santa Clara, CA, USA) following the manufacturer's instructions. pET28a-famC1 plasmid constructed as described previously [1] served as a template and the sequences of mutagenesis oligonucleotides are as follow (mutant sites are highlighted): E95A, 5'-GGTACCCAGTTTTATAATCAGCTGGCCCCG**GCA**GGCCGTAATATTG-3'; N98A, 5'-TTTTATAATCAGCTGGCCCCGGAAGGCCGT**GCA**ATTGGTTATATCT-3'; N137A, 5'ACCAAATATACCCTGACCGTTGATGTTGGT**GCA**CTGGCAGGTACCT-3'; D216A, 5'-CTGCAGGATAAATTTTCTGGTCTGGATTTT**GCA**AACGTGCGTCTGA-3'; E39A, 5'-CCGATTAATAATGCCGGCTTT**GCA**AATCCGTTTATGGATGTGGTT-3'; E175A, 5'-CATAATAATCTGTTTATTAAG**GCG**GGTGAGTTTAAAACCAGTACC-3'. N137A/E175A double mutant was constructed by introducing E175A mutant to the N137A singlet. Similarly, E39A/N95A/E98A/D216A was constructed by introducing individual mutation to the single, double, and eventually triple mutants. The recombinant plasmids were verified by sequencing.

Expression and purification of recombinant FamC1 variants

The plasmids carrying FamC1 variants were transformed into *E. coli* BL21(DE3)

cells and the protein expression were induced by 0.2 mM isopropyl β-D-1-thiogalactopyranoside (IPTG) at 16 ºC for 20 hours. Protein purification was carried out at 4 ºC. Cells were harvested by centrifugation at 5,000 x g for 15 minutes and re-suspended in lysis buffer containing 25 mM Tris-Cl [pH 7.5], 150 mM NaCl and 20 mM imidazole followed by disruption with a French Press. Cell debris was removed by centrifugation at 17,000 x g for an hour. The supernatant was then applied to a Ni-NTA column with FPLC system (GE Healthcare) and eluted by using a 20-500 mM imidazole gradient. The target protein was eluted at ~150 mM imidazole. Fractions containing the target protein were pooled and then dialyzed against 25 mM Tris-Cl [pH 7.5]. The protein solution was loaded onto a DEAE Sepharose column and eluted by using a 0-500 mM NaCl gradient. Target protein was eluted at ~150 mM NaCl. The purified protein was concentrated to 10 mg/mL in 25 mM Tris-Cl [pH 7.5] buffer and stored at -80 ºC. Protein purity was verified by SDS-PAGE analysis.

Enzyme activity measurement

The enzymatic activity of wild type and mutant FamC1 was measured in a one-pot reaction which comprises two-enzyme reaction system as described previously.[1] Briefly, 100 μl reaction containing 1 mM *cis*-indole isonitrile, 1 mM GPP, 5 μM FamD2, and 10 μM wild type or mutant FamC1, 5 mM $MgCl_2$, 5 mM $CaCl_2$, and 50

mM Tris [pH 7.8] was incubated at 37 ºC for an hour. The reaction was quenched and extracted with an equal volume of ethyl acetate. The organic fractions were dried and re-dissolved in 500 μl acetonitrile for HPLC analysis by using Agilent Extend-C18 5 μm 4.6 x 250 mm column and a solvent system of MeCN and H_2O (0.1% formic acid). The peak area of cyclized product of each sample was calculated and presented as a percentage of the wild type enzyme. All samples were analyzed in triplicate in each independent experiment.

Crystallization, data collection, structure determination and refinement

Sitting-drop vapor diffusion method was used for crystallization of FamC1 variants. In general, 1 μL protein (10 mg/mL) was mixed with 1 μL of reservoir solution in 48-well Cryschem Plates, and equilibrated against 100 μL of the reservoir at 25 °C. FamC1 N137A crystal was obtained in the crystallization buffer containing 12 % - 18 % PEG3350, 2.5 % - 3.8 % tryptone and 0.05 M HEPES [pH 7.0]. Crystal was soaked in a cryo-protectant containing mother liquid and additional 15 % glycerol and mounted in a cryo-loop and flash-cooled by liquid nitrogen for data collection. Data sets were collected at beam lines BL15A1 of the National Synchrotron Radiation Research Center (NSRRC, Hsinchu, Taiwan) and processed by using HKL2000.[2] Prior to structure refinement, 5 % randomly selected reflections were set aside for calculating R_{free}[3] as a

monitor of model quality. The phase information of the structures of FamC1 N137A was solved by using molecular replacement program Phaser[4] in CCP4i suit[5] using crystal structure of apo form FamC1 (PDB ID, 5YVK). All structural refinements were carried out using Phenix[6] and Coot.[7] All figures were prepared by using the PyMOL program (http://pymol.sourceforge.net/).

Figure Legends:

Figure S1. Stereo-views of electron density maps of Ca-coordinating residues in N137A structure. Residues in N137A variant that coordinate to (**A**) Ca1 and (**B**) Ca2 in wild type FamC1 are depicted. The $2F_o$-F_c electron density maps of these residues and Ca2 are contoured at 2.0 σ. The residues and calcium ion are shown in stick and sphere models. The A137 residue is highlighted in magenta.

Figure S2. Overall structures (**A**) and stereo-view of structure superimposition (**B**) of N137A and wild type FamC1. The overall structures of N137A and wild type FamC1 are displayed in green and cyan cartoon models, respectively. Ca^{2+} ions and CI-4 are displayed in sphere and stick models.

Figure S3. Conformation of D214-Y89 pair of Stig cyclases. D214 and Y89 from various polypeptide chains of apo-form structures and CI-4 (stick models) bound complex structures of FilC1 and HpiU5 are shown in line models. Y89 in A- and B-pose are indicated.

Scheme:

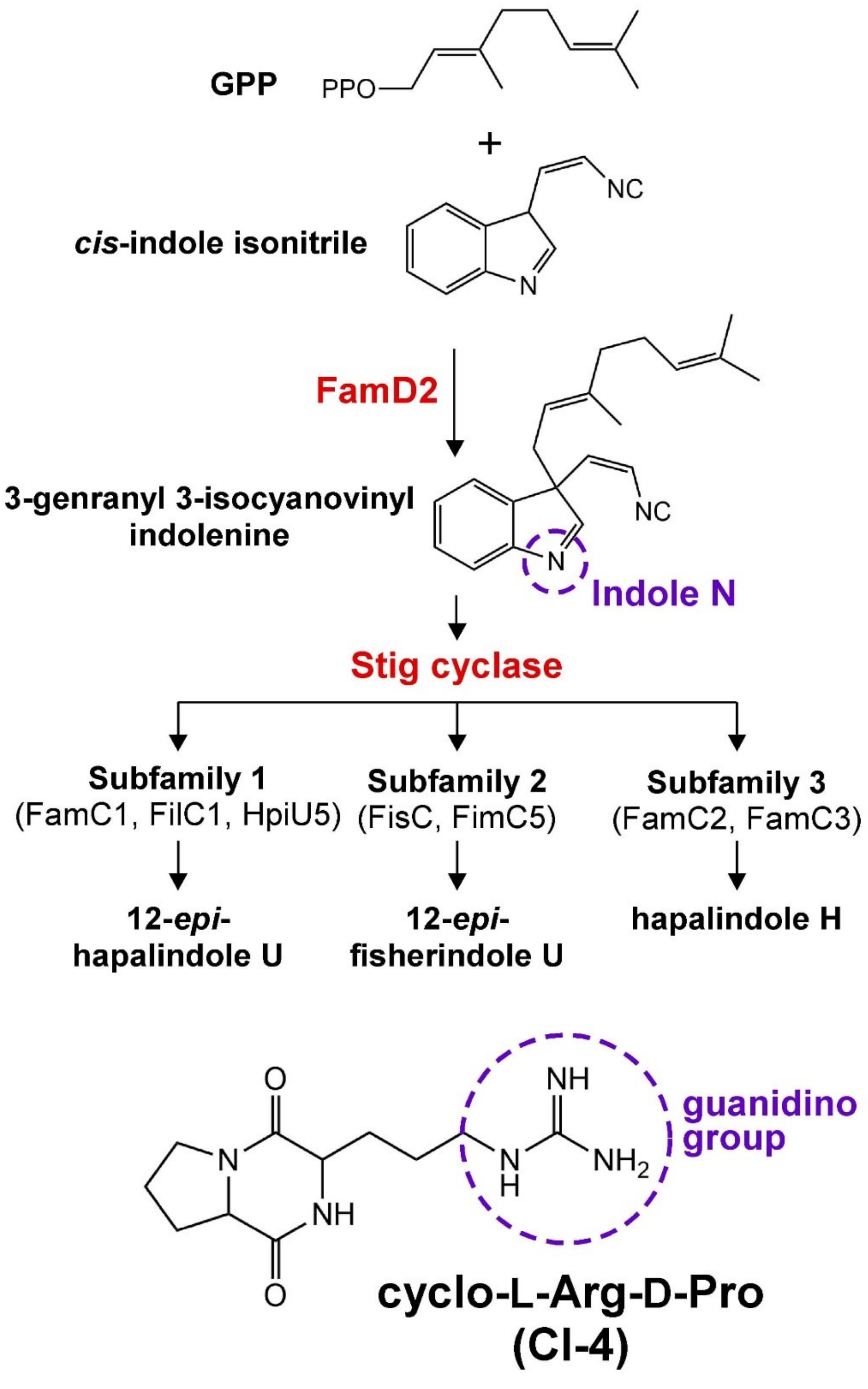

Scheme 1 Biosynthetic pathway of some hapalindole-type alkaloids and chemical structure of cyclo-dipeptide CI-4.

Figures:

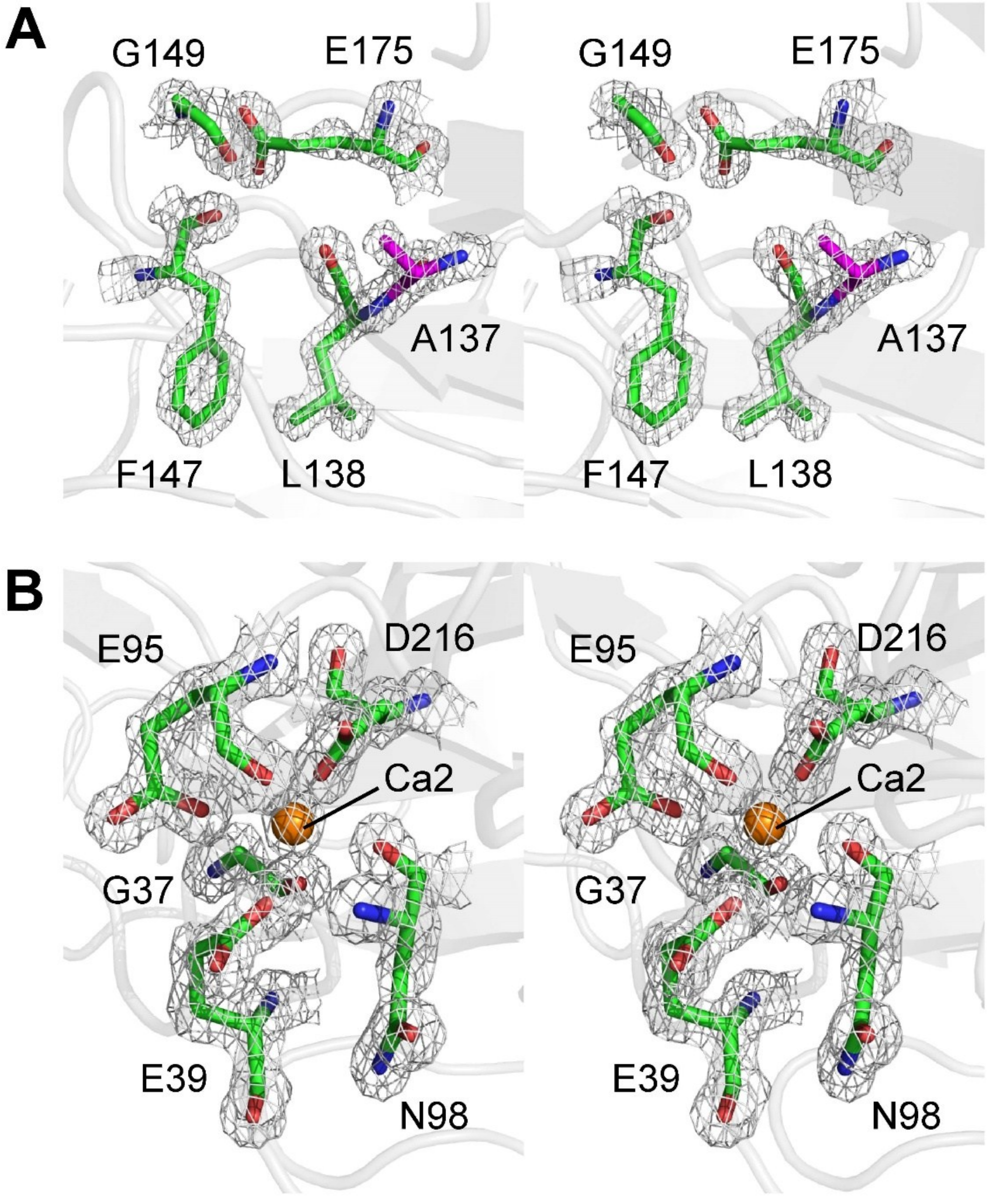

Figure S1

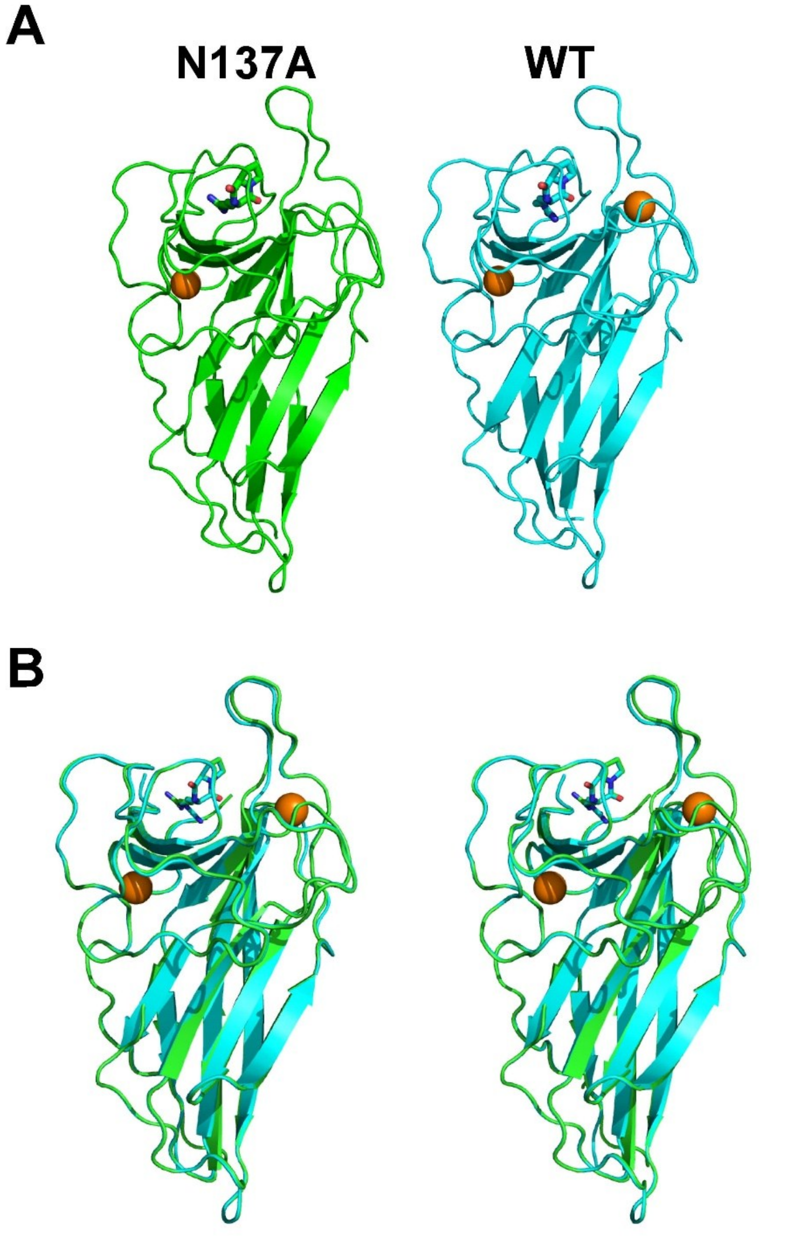

Figure S2

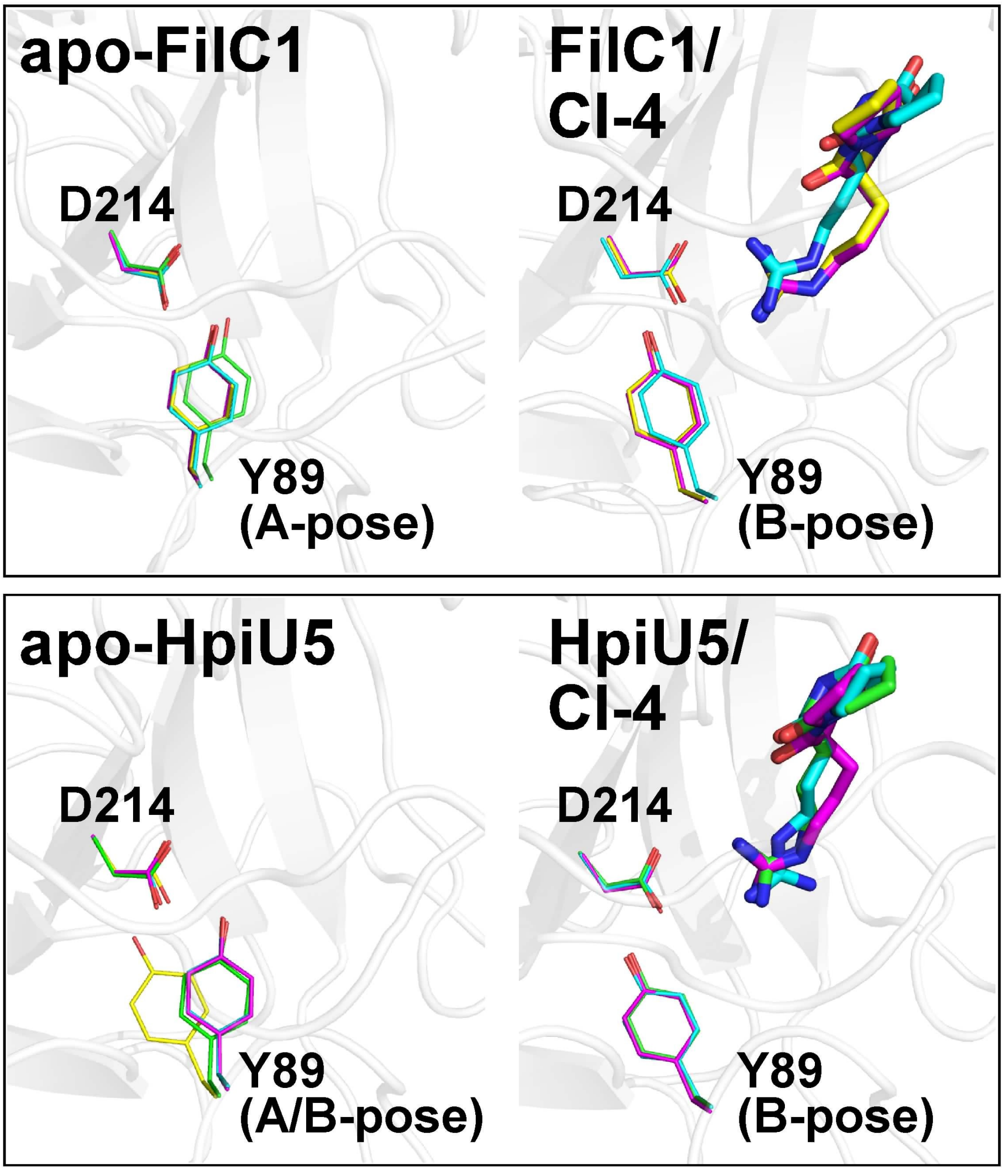
apo-FilC1
D214
Y89
(A-pose)
FilC1/
Cl-4
D214
Y89
(B-pose)
apo-HpiU5
D214
Y89
(A/B-pose)
HpiU5/
Cl-4
D214
Y89
(B-pose)

Figure S3

Table

Table S1. Data collection and refinement statistics for FamC1 N137A crystal

**Data collection**	
space group	$P3_121$
unit-cell [Å]	
a/*b*/*c*	54.78/54.78/152.42
α/*β* /*γ* (°)	90/90/120
resolution [Å]	25 – 1.48<br>(1.53 –)
unique reflections	44964 (4314)
redundancy	3.8 (3.0)
completeness [%]	99.5 (98.2)
average I/σ (I)	34.1 (3.4)
R_{merge}	3.7 (20)
R_{pim}	2.2 (13.3)
CC1/2	0.986 (0.938)
**Refinement**	
no. of reflections	44915 (3165)
R_{work} (95 % of data)	10.8 (15.8)
R_{free} (5 % of data)	14 (18.3)
r.m.s.d. bonds [Å]	0.010
r.m.s.d. angles [º]	1.441
MolProbity statistics	
Clashscore	4.8
Ramachandran favored [%]	98.99
Ramachandran allowed [%]	1.01
Ramachandran outlier [%]	0.00
MolProbity score	1.43
no. of non-H atoms / average B [Å^2]	
Protein	1565/18.6
Water	331/35.3
Ligand	19/14.1
PDB ID	6J03

Values in parentheses are for the highest resolution shell.

Reference

1. C. C. Chen, X. Hu, X. Tang, Y. Yang, T. P. Ko, J. Gao, Y. Zheng, J. W. Huang, Z. Yu, L. Li, S. Han, N. Cai, Y. Zhang, W. Liu and R. T. Guo, *Angewandte Chemie (International ed. in English)*, 2018, **57**, 15060-15064.
2. Z. Otwinowski and W. Minor, *Method in Enzymology*, 1997, **276**, 307-326.
3. A. T. Brunger, P. D. Adams, G. M. Clore, W. L. DeLano, P. Gros, R. W. Grosse-Kunstleve, J. S. Jiang, J. Kuszewski, M. Nilges, N. S. Pannu, R. J. Read, L. M. Rice, T. Simonson and G. L. Warren, *Acta Crystallogr D Biol Crystallogr*, 1998, **54**, 905-921.
4. A. J. McCoy, R. W. Grosse-Kunstleve, P. D. Adams, M. D. Winn, L. C. Storoni and R. J. Read, *J Appl Crystallogr*, 2007, **40**, 658-674.
5. M. D. Winn, C. C. Ballard, K. D. Cowtan, E. J. Dodson, P. Emsley, P. R. Evans, R. M. Keegan, E. B. Krissinel, A. G. Leslie, A. McCoy, S. J. McNicholas, G. N. Murshudov, N. S. Pannu, E. A. Potterton, H. R. Powell, R. J. Read, A. Vagin and K. S. Wilson, *Acta Crystallogr D Biol Crystallogr*, 2011, **67**, 235-242.
6. P. D. Adams, P. V. Afonine, G. Bunkoczi, V. B. Chen, I. W. Davis, N. Echols, J. J. Headd, L. W. Hung, G. J. Kapral, R. W. Grosse-Kunstleve, A. J. McCoy, N. W. Moriarty, R. Oeffner, R. J. Read, D. C. Richardson, J. S. Richardson, T. C. Terwilliger and P. H. Zwart, *Acta Crystallogr D Biol Crystallogr*, 2010, **66**, 213-221.
7. P. Emsley and K. Cowtan, *Acta Crystallogr D Biol Crystallogr*, 2004, **60**, 2126-2132.
